# Cognitive Biases and Socio-Occupational Functioning Mediate the Relationship between Executive Functions and the Severity of Psychopathology among Young Adults with Psychotic-like Experiences: 1-Year Follow-Up Study

**DOI:** 10.3390/brainsci14030256

**Published:** 2024-03-05

**Authors:** Aleksandra Arciszewska-Leszczuk, Andrzej Cechnicki, Dorota Frydecka, Dawid Kruk, Łukasz Gawęda

**Affiliations:** 1 Faculty of Psychology, University SWPS, 81-745 Sopot, Poland; 2 Department of Community Psychiatry, Jagiellonian University Medical College, 31-115 Krakow, Poland; acechnicki@interia.pl (A.C.); dawid.kruk@doctoral.uj.edu.pl (D.K.); 3 Department of Psychiatry, Wroclaw Medical University, 50-367 Wroclaw, Poland; dfrydecka@gmail.com; 4 Experimental Psychopathology Lab, Institute of Psychology, Polish Academy of Sciences, 00-378 Warsaw, Poland

**Keywords:** executive functions, Trail Making Test, cognitive biases, social and occupational functioning, risk of psychosis, psychotic-like experiences

## Abstract

The aim of this study was to investigate whether Trail Making Test (TMT) performance is associated with the severity of psychopathological symptoms related to psychosis among young adults with elevated level of psychotic-like experiences (PLEs), and whether this relationship is mediated by cognitive biases and socio-occupational functioning. A total of 187 subjects from a larger population of 6722 young adults participated in this 1-year follow-up study. The inclusion criteria were an elevated level of PLEs (the highest score of the Prodromal Questionnaire) and a lack of schizophrenia diagnosis. Eventually, 134 subjects (71.6%) completed the TMT, as well as the DACOBS scale (cognitive biases), at baseline and were examined twice using the CAARMS (psychopathology) and SOFAS (socio-occupational functioning) scales. In the first (I) and second (II) measurements, the calculated effects indicate indirect-only mediations, which explained 35 and 38% of the variance of the CAARMS. The TMT B execution time was positively associated with the DACOBS scale (β = 0.19, *p* = 0.028), which was negatively related to the SOFAS I (β = −0.37, *p* < 0.001) and SOFAS II (β = −0.20, *p* = 0.016) measurements. A lower score on the SOFAS I predicted a higher score on the CAARMS I (β = −0.50, *p* < 0.001), and a lower SOFAS II predicted a higher score on the CAARMS II (β = −0.61, *p* < 0.001). Subtle EF dysfunctions may, over time, translate into a greater severity of symptoms related to psychosis in people with elevated PLEs, and this is mediated by a deterioration of their metacognition and socio-occupational functioning.

## 1. Introduction

Over the last few decades, the phenomena of a categorical approach and “Kraepelinian” dichotomy [1] toward psychosis have been systematically replaced by a dimensional perspective. This new approach assumes a continuum of psychotic experiences and that symptoms are aligned between clinical and non-clinical populations [2]. This resulted in the examination of psychotic symptoms in subjects with schizophrenia-spectrum disorders and with other mental illnesses; measuring psychotic-like experiences (PLEs) in non-help-seeking individuals from the general population has also become popular [3].

PLEs refer to subthreshold psychotic symptoms in the general population, such as delusion-like thoughts and perceptual aberrances, leading to distress and impairment, but which are not synonymous with a diagnosis of schizophrenia [4]. While PLEs can manifest in healthy individuals, they may correlate with adverse health and social consequences (see the systematic review of Kaymaz et al. [5]). Individuals experiencing PLEs are at an elevated risk of developing various mental health conditions later in life, including schizophrenia (see the meta-analysis of Healy et al. [6]). Moreover, they often require greater healthcare support [7] and are at a high risk of engaging in suicidal behaviors (see the meta-analysis of Yates et al. [8]). This highlights the utility of PLEs as indicators of adverse mental health outcomes, as confirmed by the latest meta-analysis by Staines et al. [9].

Focusing on psychotic disorders, prior to their first episode, most individuals experience such subclinical symptoms like PLEs and these may be related to an ultra-high risk (UHR) state [10] or at-risk mental state (ARMS) [11]. This “at-risk” group is defined as meeting the criteria for one or more of three syndromes: (1) attenuated positive symptoms (APS) syndrome, (2) brief limited intermittent psychotic symptoms (BLIPS) syndrome, or (3) genetic risk and/or deterioration (GRD) syndrome [12]. An UHR state is linked to a 22% risk of developing a full-blown psychosis within 3 years [13]. Moreover, most of the UHR/ARMS individuals who do not develop a psychotic disorder continue to exhibit subthreshold symptoms or meet the criteria for other mental illnesses [14,15,16]. In addition, functioning impairment is prevalent in these groups [13], as well as among individuals with PLEs [5]. One manifestation of this functional deterioration may be cognitive decline and its consequences, which will be discussed further.

Neurocognitive impairment, understood as a deficit in various cognitive functions (attention, working memory, verbal and visual learning, processing speed, executive functions and social cognition), is a core feature of psychosis [17]. In different meta-analyses, prospective studies investigating individuals exhibiting clinical [18,19] and genetic [20] risk states for psychosis consistently illustrate that cognitive decline emerges early in the disease course. Moreover, individuals at risk of psychosis, who later transitioned to psychosis, exhibit greater cognitive impairments at baseline compared to those who do not develop full-blown illness [18,19]. These findings are also supported by retrospective studies on the premorbid IQs of patients diagnosed with schizophrenia [21]. Cognitive deficits can thus be viewed as inherent characteristics of the “at-risk” group and should be continuously analyzed.

Executive function (EF) decline represents one of the most frequent neurocognitive impairments in the schizophrenia spectrum [22,23]. In addition, it is identified not only in chronic patients [24], but also prior to the onset of the disorder [25,26,27]. The impairment is often described as problems with conceptualization, planning, cognitive flexibility, verbal fluency, solving complex problems and the ability to quickly and efficiently shift back-and-forth between mental sets [22].

Executive dysfunction is an independent predictor of both the transition to psychosis and the functional outcomes within the UHR population [28]; however, research results are not consistent. For instance, the study of Albert et al. [29] revealed that worse childhood executive functions (EFs) are predictive of greater prodromal symptoms of psychosis in young adulthood. Also, in the retrospective cohort study of Rembark et al. [30], baseline executive function impairment was associated with a schizophrenia diagnosis and psychiatric treatment at follow-up. At the same time, in another study [17], executive functions show the largest impairments in different “at-risk” groups, although they do not predict a later transition to psychosis.

However, what is crucial is that, among subjects at risk of psychosis, executive functioning deficits at a baseline assessment can serve as a significant predictor of overall social functioning and its later decline. Work role impairment is also accounted for by deficits in executive functioning [31]. As one can see, this domain is primarily important for the global functioning of individuals. It may not necessarily, especially not in the first place, have prognostic value (in terms of assessing the likelihood of developing full-blown psychosis). For this reason, this area warrants attention (the possible consequences of EF deficits and their effect on different aspects of personal functioning will be discussed later).

Although executive functions are very important to patients’ mental states, they are also challenging to assess. They are seen as separate cognitive domain, but one consisting of different, interrelated cognitive functions. One of them is set-shifting, which refers to the ability to move back and forward between different tasks or mental sets (which is related to cognitive flexibility). Impairments in set-shifting are seen as core EF deficits in psychosis [32,33]. Kumbhani et al. [34] reported the largest impairments in this domain in patients diagnosed with schizophrenia. In general, many studies indicate that in people with psychosis, but also with psychosis proneness, flexibility and initiation may be some of the primary areas of EF’s deficits in daily life [35,36,37,38]. The same conclusions are drawn from studies on subjects with psychotic-like experiences (PLEs) [38,39].

One of the most frequently used neuropsychological tests that measures the efficiency of executive functions is the Trail Making Test—TMT (especially version B, which is an extensively used trail for the assessment of cognitive flexibility [40]). Many studies show a decline in the score of this test among patients with psychotic disorders [41,42], suggesting deficits in, i.a., set-shifting. Set-shifting facilitates the development of efficient behavior and adaptive responses to the changing circumstances of various conditions. Therefore, this ability appears to be crucial for other aspects that may contribute to the exacerbation of psychopathology among individuals on the psychosis spectrum.

The cognitive model of psychosis suggests that psychotic symptoms may arise because of biased information processing [43] and metacognition deficits [44]. EFs are one of the crucial aspects of metacognition [45], which is understood as “cognition about cognitive phenomena” [46] (p. 906). What is important is that cognitive shifting (set-shifting) and mental flexibility are two of the basic cognitive functions in metacognition.

A poor metacognitive self (MCS), defined as a decreased self-awareness and understanding of one’s own thought processes, may lead to cognitive biases [47]—systematic patterns of deviation in judgment, in which the inferences made about other people and/or situations can be illogical [48]. Such cognitive biases are associated with the schizophrenia spectrum [49,50] and often manifest as jumping to conclusions (making judgments without a sufficient amount of information) and belief inflexibility, external attribution bias, or paying attention to threats (excessive perception of others’ behavior as threatening) [51]. They could be also social cognition problems [52]

Many studies have confirmed that exaggerated cognitive biases are observed along the continuum of psychosis from psychotic-like experiences (PLEs) to full-blown psychosis [50]. In addition, besides being one of the characteristics of psychosis, cognitive biases play an important role in the appearance of psychotic symptoms, e.g., hallucinations and delusions—even among healthy individuals [53,54]. The study of Hakamata et al. [42] also indicated that the general attentional ability (as well as working memory and set-shifting) measured by the TMT is related to, e.g., the attentional bias in the non-clinical population, which suggests that deteriorated executive functions may contribute to poorer metacognitive skills. This last conclusion is the result of many studies on patients with psychotic disorders and, among others, Davies et al. [55] suggest that “metacognition may be critical to translating cognitive and functional skills into real-world contexts at early stages of illness” (p. 824).

Both poor executive functions and the presence of cognitive biases are significant predictors of the deterioration of emotional, social and professional functioning. As it was shown in the context of EFs, set-shifting seems to be significant, as well as working memory and processing speed. Such dysfunctions may affect the clinical course of illnesses [56,57,58] and also predict social functioning difficulties not only among psychotic patients [59] but also in the UHR group [60]. In papers by Eslami et al. [31] and Meyer et al. [61], in the “at-risk” groups these difficulties were associated with executive dysfunction (including set-shifting impairment).

As shown in other studies, poor EF (including poor set-shifting), manifesting as difficulties in time management and multitasking, memory and concentration deficits, or organizational problems, may also have a crucial impact on a person’s performance at work [62,63,64]. Among patients with schizophrenia, executive dysfunction has been found to be related to their employment status and job performance [65,66]. In the longitudinal study of Lam et al. [67], changes in attention and executive function (like set-shifting) accounted for the social and occupational functioning of UHR individuals. Although there are no specific studies on employment, cognitive difficulties and the severity of psychotic-like experiences, some developmental markers have been identified in samples of children and young adolescents reporting PLEs, including language processing difficulties [68] and early speech and/or motor delays [69], as well as EF deficits (including set-shifting impairments) [70]. These may affect their future performance at school [71], and, consequently, at work.

Importantly, social problems and poor job performance have a negative impact on mental health [72,73] and, as a cause of chronic stress, may result in growing psychopathological symptoms (cf. stress–vulnerability model [74,75,76]). As mentioned earlier, both executive dysfunction and cognitive biases can lead to socio-occupational dysfunction as well as to psychopathological symptoms. The question remains as to how these last two variables are related to each other. It is assumed that, among patients diagnosed with schizophrenia, the severity of symptoms affects functional impairment [77,78,79]. However, this association may not appear universal, particularly in individuals who have not yet developed psychosis but are experiencing PLEs. As it has not been explored in detail before in this particular study group, it is also crucial to analyze how some executive deficits may contribute to the worsening symptoms associated with psychosis and what role cognitive biases play.

Given the above, the aim of this study was demonstrate the important (potentially mediating) role of cognitive biases and social and occupational functioning in the relationship between the executive aspects of cognition and psychopathology among people experiencing psychotic-like experiences. Hence, we tested a serial mediation model in which a greater number of cognitive biases and poor social and occupational functioning will mediate the relationship between impaired set-shifting (a poor TMT performance) and the global severity, as well as frequency, of various psychopathological symptoms (which are associated with a high risk of psychosis). Moreover, we investigated whether this dependency would persist during a 12-month follow-up. We hypothesize that, in this study group, poor set-shifting that leads to cognitive biases (mediator 1) may predict and cause the continuance of decreased everyday functioning (mediator 2) and thus the increase severity and frequency of psychopathological symptoms a year later. What is important is that such a model allows for the investigation of the earliest cognitive factors associated with psychosis proneness, while excluding potential confounding factors such as disease chronicity and antipsychotic use.

## 2. Materials and Methods

### 2.1. Participants

A total sample of 134 young adults aged 18 to 35 (56.7% women) were included in our longitudinal study (while the literature defines the at-risk group for schizophrenia as individuals aged 15–25 years, we opted not to include individuals under the age of 18 due to the challenge of obtaining parental consent for this study. Furthermore, the age range of 18–35 appeared to overlap with other studies conducted in the general population and even among individuals experiencing their first episode of a psychotic disorder (see, e.g., [80,81]). This group came from a larger population of 6264 young adults who participated in a project on PLEs, completing the online survey of a Computer Assisted Web Interview (CAWI) [82]. Based on their PQ-16 (Prodromal Questionnaire) value (range 0–48, with a cutoff score of 6), 187 participants with the highest scores (with an elevated level of PLEs) were invited to a face-to-face meeting with mental health professionals and examined using a comprehensive set of various clinical and psychological tests (to be more clear: those who scored within 7% of the top results on the PQ-16 (scores 6–48) and met the inclusion criteria were approached to participate in the face-to-face assessment. We planned to recruit approximately 200 participants from approximately 6000 subjects (3.3% of the sample studied) who achieved scores on the PQ-16 that were within the top 10%. We chose a wider percentage of the highest scores to recruit from, expecting that not all participants would meet the inclusion criteria and would be willing to take part in the second stage of the study. Finally, we personally examined 187 people whose results on the PQ-16, with respect to the entire sample, turned out to be the top 7%).

This research model assumed repeated measurements (at the beginning and after 1 year), but 53 people did not show up at the second meeting. Finally, 134 subjects (71.6% from *n* = 187) who agreed to take part in the study and gave their informed consent completed all scales. The exclusion criteria were a diagnosis of any psychotic or neurological disorder, taking antipsychotic medication, or a substance use disorder in the past 6 months (which was verified during their interview with a qualified psychiatrist). We decided to exclude the aforementioned individuals to reduce the potential confounding effect of the psychotic symptoms occurring in schizophrenia or resulting from conditions related to intoxication or central nervous system damage. Including other participants (with other mental illnesses) in the study allowed us to avoid further reduction in the sample size.

The study was approved by the Ethics Committee of the Medical University of Warsaw.

In general, the research group comprised young individuals exhibiting an elevated level of PLEs (*M* = 22.9 in the Prodromal Questionnaire with a cut-off score of 6). Among them, a portion (20.9%) had received diagnoses of conditions other than psychotic disorders (primarily depression). These diagnoses were self-reported and subsequently confirmed by a mental health professional during their initial meeting. A majority of participants (over 60%) expressed the necessity of seeking help from a psychologist or psychiatrist due to the emotional distress they had experienced. Additionally, 34% of the participants had a positive family history of mental illness, predominantly involving a parent.

Considering the correlation between PLEs and psychoactive substances (as outlined in the meta-analysis by Matheson et al. [83]), we also gathered information regarding their usage among the subjects. It was revealed that the most commonly used substance was alcohol (88%), followed by marijuana or hashish (45.5%). Nearly one fifth of the participants also reported using amphetamine.

Detailed sociodemographic and clinical data on the analyzed sample can be found in Table 1 and Table 2, along with basic information on the dropout group (*n* = 53).

As can be seen, the groups did not differ in any sociodemographic characteristics. As for clinical data, more personality disorders and a more frequent use of psychotropic drugs, as well as marijuana and amphetamine, were reported in the dropout group (Table 2).

### 2.2. Assesments

To evaluate the factors described in the introduction, we used the previously mentioned Trail Making Test (TMT) which enabled us (taking into account the time-consuming nature of the face-to-face examinations) to quickly assess patients’ simple set-shifting ability (as a measure of their executive functions). Additionally, we used the DACOBS scale to examine cognitive biases (especially those typical of psychosis), as well as two widely-used psychiatric scales: SOFAS, to assess social and occupational functioning, and CAARMS, a semi-structured interview to evaluate the severity of symptoms related to the risk of psychosis.

#### 2.2.1. TMT—Trail Making Test

The TMT is a connect-the-dot task from the Halstead–Reitan Neuropsychological Battery [84]. The efficacy of this test lies in its ability to distinguish brain-damaged patients from normal control subjects with a relatively high degree of accuracy [85,86].

The TMT-A is essentially a test of processing speed and requires the connection, in sequence, of 25 dots labeled with numbers as quickly as possible. The TMT-B is a combination of processing speed and mental flexibility/switching ability and requires the connection, in sequence, of 25 dots labeled with alternating numbers and letters, in which the tempo is also measured. The longer the time taken in part A, the lower the psychomotor speed; the longer the time taken in part B, the weaker the mental flexibility. In the study of Arbuthnott et al. [87], TMT-B performance was associated with set-switching. Thus, the assumption underlying our clinical interpretation of the test appears to be valid, and the test may be confidently used to assess executive function.

This tool was only used at the baseline measurement.

#### 2.2.2. DACOBS—Davos Assessment of Cognitive Biases Scale

The DACOBS scale is a self-report questionnaire that allows us to measure both the cognitive biases accompanying mental disorders (especially psychotic disorders) and cognitive deficits. The subscales of the DACOBS are intercorrelated, with many validation tests such as the beads task, Green Paranoid Thoughts Scale, or Dogmatism Scale. According to author of the tool, it is reliable and valid for use in clinical practice and research [88].

In this study, the Polish translation prepared by Gawęda et al. [89] was used. The scale consists of 42 items. The respondent is asked to indicate to what extent a given statement reflects their way of thinking. The scale has seven degrees, from I strongly agree to I strongly disagree. The results obtained constitute the total score (analyzed in this study), which is a measure of the global severity of cognitive biases, which refer to the jumping to conclusions bias, belief inflexibility bias, attention to threat bias, external attribution bias, social cognition problems, subjective cognitive problems and safety behaviors. The original validation studies indicate a high reliability of the questionnaire—Cronbach’s alpha is 0.90 for the general scale [88]. In this study, a satisfactory result of 0.88 was obtained and the tool was only used at the baseline measurement.

#### 2.2.3. SOFAS—Social and Occupational Functioning Scale

The SOFAS is the clinician-rated scale from 0 to 100 initially described in a paper by Goldman et al. [90]. The tool is commonly used to assess the level of functioning in patients with schizophrenia [91], although researchers also correlate its results with, e.g., PLEs [92,93]. It has been shown to have good face and construct validity [94]. The SOFAS indicates the level of social and occupational functioning of a person over a recent period. It refers to a continuum, ranging from a state of optimum functioning (80–100) to the state of worst functional impairment, without taking symptoms into account and regardless of the cause of impairments [95].

In this study, the tool was used twice (at the baseline measurement and during the 1-year follow-up).

#### 2.2.4. CAARMS—Comprehensive Assessment of At-Risk Mental States

This is a semi-structured interview designed to identify individuals who are at clinical ultra-high risk (UHR) of developing psychosis [96]. The CAARMS includes the following seven subscales: 1—positive symptoms, 2—cognitive change in attention/concentration, 3—emotional disturbance, 4—negative symptoms, 5—behavioral change, 6—motor/physical changes and 7—general psychopathology. Within each group, individual symptoms and the questions useful for identifying them are listed. Then, the severity of the symptoms is assessed on the following scale: 0—symptom absent, 1—doubtful, 2—mild, 3—moderate, 4—moderately severe, 5—severe and 6—depending on the symptom: extreme or psychotic. After determining the severity of the symptom, its frequency and duration should be assessed. Therefore, for all scales, an indicator of general severity and general frequency can be calculated (the higher the measure, the more severe and more frequent the symptoms). The CAARMS is widely used both in clinical practice and in research on psychosis risks [97]. The reliability and validity of this instrument were confirmed by Yung et al. [96]. It shows excellent inter-rater reliability when performed by trained raters (0.85) [98].

In this study we used the Polish translation [99]. We conducted a full interview twice and, for the purposes of this study, we focused on the global approach and used the sum of the general severity and general frequency scales. Contrary to the recommended CAARMS scoring system that enables the evaluation of clinical criteria for ARMS, we were interested in a global severity index of symptoms. Hence, according to previous work (e.g., [82]), we combined the total scores for the frequency and intensity subscales, which resulted in a total score of the severity of symptoms.

The tool was used twice (at the baseline measurement and during the 1-year follow-up).

### 2.3. Data Analyses

All statistical analyses were performed with SPSS version 25.0. First, we assessed the distributions of the variables using a Shapiro–Wilk test and checked skewness, assuming that values >−2 or <2 confirm a normal distribution of the quantitative data [100]. When analyzing the differences between the study group and dropouts (see Table 1 and Table 2), we used the Mann–Whitney U-test (which was dictated by the significant difference in group sizes) along with the chi-square test and Fisher’s exact test.

In our main analysis, we initially conducted a series of zero-order correlations to investigate the associations between quantitative variables. In order to calculate the serial mediation models, we used PROCESS macro ver. 4.0 for SPSS and chose model 6 [101], following the bootstrapping procedure with 5000 resamples. Ultimately, two models were built (A and B). In model A, we analyzed the relationship between the TMT execution time (independent variable) and CAARMS scores (dependent variable)—both measured at the baseline. In this analysis we also included two mediators—the total score of the baseline DACOBS (the first mediator) and SOFAS (the second mediator). Therefore, the whole model tested the relationship between the levels of the variables in the first measurement; at one time point. In Model B, we wanted to look at the relationship between TMT performance (measured at the baseline) and CAARMS scores after a one-year follow-up. We also added two mediators to this analysis—the DACOBS results (the first mediator) from the first measurement and the SOFAS results (the second mediator) from the follow-up measurement. In this way, we checked how the initial level of executive function and cognitive biases are related to the results of socio-occupational functioning and the severity of psychopathology after a year.

## 3. Results

### 3.1. Differences between the Study Group and Dropouts

The groups did not differ in terms of any of the questionnaire variables analyzed in this study (see Table 3).

### 3.2. Data Distributions

The Shapiro–Wilk test indicated that almost all of the variables in final study group were not normally distributed (*p* < 0.05). However, due to the relatively large population and the skewness of all study variables, which were between >−2 and <2 [100], we assumed that the sampling distribution would not differ from a normal distribution and, therefore, that the use of parametric tests would be allowed.

### 3.3. Correlational Analyses

The Pearson’s correlation analysis with FDR correction for multiple comparisons (Table 4) indicated that it was the TMT B execution time that correlated with almost all of the other results (what is the most important is that this variable was associated with the first mediator, DACOBS, while the TMT A execution time was not); therefore, this variable was included in the mediation models. The table also shows that the DACOBS, as the first potential mediator, negatively correlated with both measurements of the SOFAS scale, which was selected as the second mediator. Moreover, almost all variables correlated with both the CAARMS measurements (the exception was the DACOBS scale, which was only correlated with CAARMS I (according to the new approach to mediation analysis [101,102], the lack of a significant simple correlation of the mediator with the dependent variable does not exclude this mediating variable from further analysis. Only the relationship between the independent variable and the mediator is necessary to test a mediation model. This is due to the fact that the impact of the mediator on the dependent variable is always analyzed simultaneously with the impact of the independent variable on dependent variable, while the relationship between the independent variable and the mediator is calculated as a simple regression (in first step of the analysis)).

### 3.4. Mediation Models

**Figure 1:**
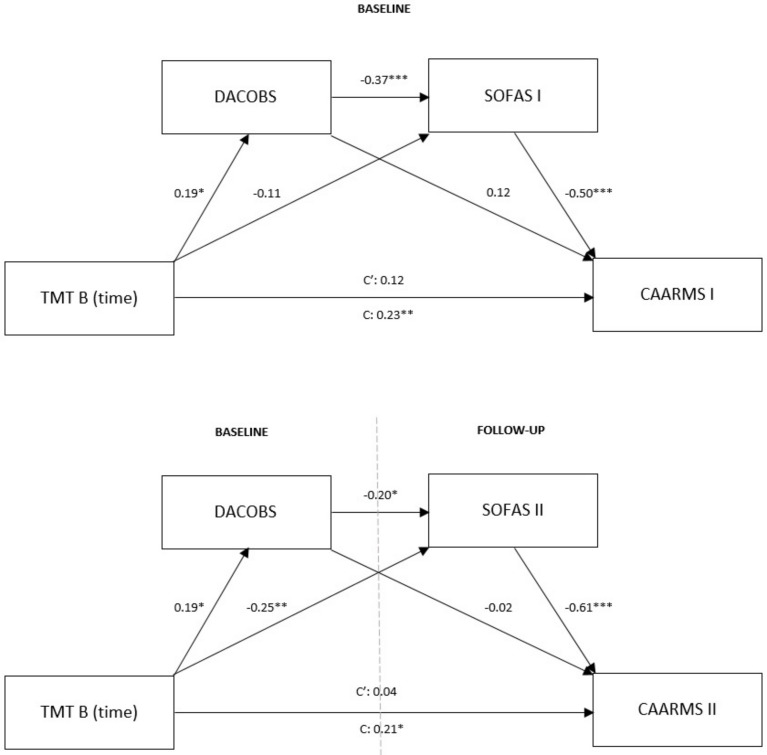
Mediation models A and B (coefficients are standardized). A probability note for *p*-values: * *p* ≤ 0.05, ** *p* ≤ 0.01, *** *p* ≤ 0.001.

To determine the serial mediation of cognitive biases and socio-occupational functioning in the relationship between TMT B execution time and the frequency and severity of psychopathological symptoms, a regression-based approach and bootstrap method, as recommended by Hayes [101], were used. The calculated models A and B (Figure 1) represent (according to the nomenclature provided by Zhao et al. [102]) indirect-only mediations.

In model A, the non-standardized total effect of the TMT B execution time and CAARMS I was significant (0.35, 95% CI: 0.10–0.61, *p* = 0.007) and it explained 5% of the dependent variable’s variance. After including both mediators, a significant indirect effect (non-standardized indirect effect: 0.17, 95% CI: 0.03–0.30) and no significant direct effect (non-standardized direct effect: 0.18, 95% CI: −0.04–0.41, *p* = 0.099) were observed, and the explained variance increased to 35%. Thus, the initial and direct relationship between the independent and dependent variable was apparent and it was explained by an indirect effect, i.e., executive dysfunction can predict an increase in the number of cognitive biases; more cognitive biases can predict a stronger decline in socio-occupational functioning, and this may lead to a greater number and severity of psychopathological symptoms.

Similarly, in model B, which included the SOFAS and CAARMS scores after one year, although initially the total effect was significant (non-standardized total effect: 0.35, 95% CI: 0.07–0.62, *p* = 0.013; R^2^ = 5%), after including the mediators and revealing a significant indirect effect (non-standardized indirect effect: 0.28, 95% CI: 0.06–0.52), the direct relationship between the independent and dependent variable disappeared (non-standardized direct effect: 0.06, 95% CI: −0.17–0.29, *p* = 0.591), and the entire model explained 38% of the variance observed.

## 4. Discussion

The aim of this study was to demonstrate the mediating role of cognitive biases and the decline in social and occupational functioning in the relationship between executive deficits (defined as a worse set-shifting ability) and the severity of psychopathological symptoms linked to the psychosis risk among people experiencing PLEs. This hypothesis was tested both at the beginning of the study and during a 1-year follow-up (taking into account potential changes in socio-occupational functioning and the severity of psychopathology).

It was found that a poor TMT B performance (suggesting set-shifting deficits) may contribute to cognitive biases and that this result is consistent with the neurocognitive perspective of psychosis [103]. This mechanism can likely be explained by the fact that impaired cognitive functioning (including a decrease in cognitive flexibility and difficulties with set-shifting) results in a deterioration of thinking ability and increased misinterpreting events. This may ultimately lead not only to a decrease in intellectual functioning, but also to lower general adaptive skills.

According to the research findings of Lysaker et al. [104], disrupted metacognition and numerous cognitive biases (arising from poor neurocognition) underlie social problems and may also contribute to a decreased job performance in individuals with schizophrenia. Empirical evidence suggests that cognitive biases affect attention, decision-making/reasoning, memory recall, motivation and even attributional style [105,106]. The tendency to making hasty decisions or the incapacity of modifying one’s own belief, as well as an attention to threats can also be noticed among people experiencing PLEs [107], and such difficulties may interfere with their everyday functioning at work or in social environments. Importantly, our study also revealed that greater cognitive biases are linked to lower levels of social and occupational functioning. Furthermore, executive dysfunction (expressed as poor set-shifting), together with the resulting cognitive biases, can predict these adverse socio-occupational outcomes even after a year. Interestingly, we observed that, even after excluding cognitive biases, TMT performance was significantly associated with socio-occupational functioning after one year, but not at the same time point. The presence of cross-correlation can imply a potential causal relationship and suggest that, in this group, some subtle executive deficits may directly influence their job performance and social skills over time (and consequently be associated with the severity of their psychopathology). This observation is consistent with the longitudinal study conducted by Lee et al. [108].

Social and occupational functioning is often diminished shortly before the outbreak of psychosis [109]. The present study also showed that a lower SOFAS score predicts a higher severity and frequency of symptoms in the CAARMS, which is consistent with the existing literature. According to the ARMS criteria defined by CAARMS [82], the decline in overall functioning (assessed by the SOFAS) is a significant symptom indicating a potential transition to full-blown psychosis, but it should be associated with the severity of the symptoms of the UHR individual. In this study, it should also be emphasized that, according to the stress–vulnerability model [74,75,76], worse functioning at work or in the social environment may increase a person’s level of stress and thus create the risk of them developing, e.g., psychosis. Such a relationship may be particularly relevant when examining individuals who do not exhibit severe psychopathology but rather only subtle symptoms (like PLEs). These symptoms may not necessarily indicate a future transition to psychosis, but, assuming that individuals with PLEs also manifest cognitive difficulties [110], e.g., poor set-shifting (which may promote cognitive biases and cause difficulties in everyday life), this may be an important factor contributing to the occurrence or intensification of the symptoms of any mental disorder. Therefore, the identification of mechanisms that could result in increased psychopathology among people with PLEs seems important and has a potential impact on improving the functioning of this population.

A novel aspect of this study was its focus on cognitive factors—particularly set-shifting ability (as a component of executive function), which is hypothesized to be associated with a greater tendency towards cognitive biases. This last variable was identified as the first significant mediating variable of psychopathological symptoms. Set-shifting and cognitive biases were also explored as predictors of a decline in socio-occupational functioning (the second mediator), which was also expected to mediate the relationship between executive functioning and the severity of psychopathology among individuals with elevated levels of PLEs.

As can be seen, certain difficulties in executive functions, particularly impaired set-shifting, may serve as predictors for the future worsening of psychopathological symptoms related to psychosis in individuals with PLEs (the potential mechanism of this relationship has been described above). Importantly, these dysfunctions, acting as precursors to cognitive biases and deteriorating general functioning, can potentially be alleviated through appropriate neuropsychological training, which could be considered a significant component of early intervention (see the literature review of Miley et al. [111], which refers to the results of studies among individuals with first-episode psychosis).

The above results remain consistent with the concepts related to the associations between cognitive functions and the course of psychotic illness [56,57,58]. However, it should be mentioned that this analysis concerns only the general population with a psychometrically elevated risk of psychosis resulting from PLEs, and not patients with a diagnosis. Nevertheless, it is crucial to pay special attention to psychotic experiences during the early stages of mental health problems, even if PLEs are not the primary reason for seeking help. Such a motivation may result, for example, from disturbing cognitive difficulties and their negative impact on the individual’s well-being and symptoms. It will then be important to take into account that cognitive biases and the individual’s level of social and professional functioning may also be important in this relationship, as they may contribute to the exacerbation of their psychopathology in future. Importantly, these factors also represent areas in which early intervention could be beneficial.

Despite providing valuable longitudinal conclusions, the study has some limitations.

First is its relatively small sample size, but this was quite representative as it was taken from a large population of subjects recruited at the screening stage. Also, we recorded a relatively high dropout rate. While these subjects did not exhibit significant differences in most key variables, they demonstrate specific features such as a higher frequency of psychoactive substance use and diagnoses of personality disorders. These aspects could potentially be related to their lower motivation to participate in the follow-up. Such individuals may also be in a poorer mental state, which suggests we might have lost important data.

Additionally, a rather unexpected observation was that the results of the CAARMS II were significantly lower than the CAARMS I. One possible reason for this is that participants who stayed in the project became more aware of their psychotic-like experiences as well as other symptoms, and they started seeking help (which resulted in a reduction in their CAARMS scores). However, it is also important to consider that symptoms may spontaneously decrease in non-clinical samples, as evidenced by recent research indicating that the majority of PLEs are transient [112]. Furthermore, the analysis of the average SOFAS results suggests that this study may have attracted high-functioning individuals. Ultimately, despite the changes in the CAARMS, the level of social functioning remained stable. This indicates the presence of other factors not considered in the study that contribute to symptom recovery. Hence, given the reference to the stress–vulnerability model, a crucial aspect of future research would involve assessing the level of stress induced by factors such as cognitive impairment or challenges in the workplace as well as in social life. According to the meta-analysis of Muddle et al. [113], stressful daily events and patients’ emotional responses to them have a significant impact on the symptoms of those with chronic psychosis, at early stage of disease as well as with subclinical PLEs. Therefore, further analyses could explore variables such as resilience [114]; coping strategies [115]; or emotional regulation, like a positive affect in response to stress [116,117], as they may play significant roles in managing and improving the symptoms in different “at-risk” mental states.

The final limitation of this study is that the authors only investigated one domain of executive function. This was a result of the extensive nature of the original study design and the necessity of keeping the number of tools to the minimum. In future research, it would be beneficial to replicate the study with a more detailed examination of the participants’ neuropsychological profiles, especially when it comes to executive functions, as the correlation between set-shifting measured by the TMT B and CAARMS scores was rather weak, which could suggest that there is a significant relationship between these general variables that could apply to another EF domain. Such an analysis could also involve exploring the associations between various executive deficits and specific cognitive biases, while evaluating their precise impact on social and work performance. It seems that this kind of research would be highly beneficial in context of coping strategies among individuals already diagnosed with schizophrenia who, despite their condition, still try to fulfill the above social and professional roles. According to the cited research, sustained work [118] and better social functioning [119] are associated with a better prognosis, especially in the early years of a disorder.

Referring to the above, this study aimed to follow the current research trend, which focuses on examining the relationship between neurocognition, metacognition, functional outcomes and symptomatology. This approach is very common in studies on schizophrenia [120,121,122,123], but is not so popular in research on non-clinical populations—and yet self-reported PLEs, even unconfirmed or transient, warrant continuous monitoring over time, particularly when reported by individuals with impaired psychosocial functioning and different reported psychiatric problems [124].

Based on this study, it appears that cognitive impairment, accompanied by cognitive biases and difficulties in social and occupational functioning, may exacerbate these aforementioned problems. Hence, despite its limitations, this analysis provides a significant foundation for further investigation into how impaired neurocognition may lead to the development of additional challenges that could escalate into more severe psychopathological symptoms among people with PLEs.

## 5. Conclusions

In this study we observed the influence of executive dysfunction on the severity of symptoms linked to the psychosis risk among individuals experiencing PLEs. However, this mechanism appeared to not be direct as it involved increasing cognitive biases, resulting in diminished social and professional functioning.

Although 75–90% of psychotic-like experiences are temporary and may cause little discomfort [15] it should be emphasized that they may be an important risk factor for a decrease in mental health. For part of the population, this risk relates to developing psychosis, but not only that. PLEs are also a risk factor for different psychiatric disorders [125,126,127,128,129,130,131]. Therefore, it is a phenomenon worth our attention, especially in the context of potential therapies aimed at other psychological difficulties that may contribute to the intensification of general psychopathology [132,133]. Hence, future research and early intervention efforts that focus on cognitive and metacognitive skills, as well as their impact on daily life, may be particularly useful in this potentially vulnerable population.

## Figures and Tables

**Table 1:** The study group’s sociodemographic characteristics and their comparison with dropouts.

	Study Group (*n* = 134)	Dropouts (*n* = 53)	Difference
Age	25.4 (±4.6); *Me* = 24	25.3 (±5.1); *Me* = 25	*Z* = −0.28, *p* = 0.783
Gender			
Women	76 (56.7%)	30 (56.6%)	ꭓ^2^ < 0.01, *p* = 0.989
Men	58 (43.3%)	23 (43.4%)	
Education			
Primary	5 (3.7%)	6 (11.3%)	FET = 7.33, *p* = 0.099
Secondary	1 (0.7%)	0 (0%)	
Vocational	58 (43.3%)	24 (45.3%)	
Incomplete higher	20 (14.9%)	11 (20.8%)	
Higher	50 (37.3%)	12 (22.6%)	
Professional situation ^a^			
Study	67 (50%)	27 (50.9%)	ꭓ^2^ = 0.01, *p* = 0.907
Work	96 (71.6%)	31 (58.5%)	ꭓ^2^ = 3.01, *p* = 0.083
Unemployed	3 (2.2%)	3 (5.7%)	*p* = 0.354 ^b^
Rent	1 (0.7%)	1 (1.9%)	*p* = 0.488 ^b^

Note: Me—median, *Z*—Mann–Whitney’s U-test, *p*—*p*-value, FET—Fisher’s exact test, ꭓ^2^—chi-squared test. a: multiple choice. b: *p*-value for FET.

**Table 2:** The study group’s clinical characteristics and their comparison with dropouts.

	Study Group (*n* = 134)	Dropouts (*n* = 53)	Difference
Psychiatric diagnosis (yes) ^a^	28 (20.9%)	16 (30.2%)	ꭓ^2^ = 1.82, *p* = 0.177
Anxiety disorder	16 (11.9%)	5 (9.4%)	ꭓ^2^ = 0.24, *p* = 0.625
Depression	19 (14.2%)	9 (17.0%)	ꭓ^2^ = 0.23, *p* = 0.628
Bipolar disorder	1 (0.7%)	2 (3.8%)	*p* = 0.194 ^b^
OCD	1 (0.7%)	0 (0%)	*p* = 1.000 ^b^
Substance use disorder	3 (2.2%)	2 (3.8%)	*p* = 0.623 ^b^
Eating disorder	3 (2.2%)	1 (1.9%)	*p* = 1.000 ^b^
Personality disorder	2 (1.4%)	7 (13.2%)	ꭓ^2^ = 11.38, *p* < 0.001
Other	3 (2.2%)	0 (0%)	*p* = 0.560 ^b^
Psychotropic drugs use (yes) ^a^	30 (22.4%)	21 (39.6%)	ꭓ^2^ = 5.69, *p* = 0.017
Antidepressive	18 (13.4%)	10 (18.9%)	ꭓ^2^ = 0.88, *p* = 0.348
Anxiolytic	17 (12.7%)	12 (22.6%)	ꭓ^2^ = 2.87, *p* = 0.090
Hypnotics	10 (7.5%)	7 (13.2%)	ꭓ^2^ = 1.52, *p* = 0.218
Other	2 (1.4%)	4 (7.6%)	*p* = 0.055 ^b^
Psychoactive substance use (yes) ^a^	120 (89.6%)	47 (88.7%)	ꭓ^2^ = 0.03, *p* = 0.862
Marijuana/hashish	61 (45.5%)	35 (66%)	ꭓ^2^ = 6.40, *p* = 0.011
Alcohol	118 (88.1%)	47 (88.7%)	ꭓ^2^ = 0.01, *p* = 0.906
Amphetamine	24 (17.9%)	18 (34%)	ꭓ^2^ = 5.62, *p* = 0.018
MDMA	12 (9%)	8 (15.1%)	ꭓ^2^ = 1.50, *p* = 0.221
Cocaine	14 (10.4%)	5 (9.4%)	ꭓ^2^ = 0.04, *p* = 0.836
Heroin	1 (0.7%)	0 (0%)	*p* = 1.000 ^b^
LSD	9 (6.7%)	8 (15.1%)	ꭓ^2^ = 3.23, *p* = 0.072
Psylocybin	9 (6.7%)	3 (5.7%)	ꭓ^2^ = 0.07, *p* = 0.791
Designer drugs	13 (9.7%)	7 (13.2%)	ꭓ^2^ = 0.49, *p* = 0.484
Other	3 (2.2%)	3 (5.7%)	*p* = 0.354 ^b^
Family history of psychiatric diagnosis (yes) ^a^	46 (34.3%)	22 (41.5%)	ꭓ^2^ = 0.85, *p* = 0.358
Parent	22 (16.4%)	9 (17%)	ꭓ^2^ = 0.01, *p* = 0.926
Siblings	10 (7.5%)	2 (3.8%)	ꭓ^2^ = 0.86, *p* = 0.354
Grandparents	9 (6.7%)	5 (9.4%)	ꭓ^2^ = 0.41, *p* = 0.525
Uncle or aunt	10 (7.5%)	4 (7.5%)	ꭓ^2^ = 0.00, *p* = 0.984
Need for psychiatric/psychological support (up to 12 months before study)			
Yes	84 (62.7%)	37 (69.8%)	ꭓ^2^ = 0.84, *p* = 0.358
No	50 (37.3%)	16 (30.2%)	

Note: *p*—*p*-value, ꭓ^2^—chi-squared test. a: multiple choice. b: *p*-value for FET (Fisher’s exact test).

**Table 3:** Questionnaire variables in study group and among dropouts.

	Study Group (*n* = 134)	Dropouts (*n* = 53)	Difference
PQ-16 (screening range 6–48)	22.9 (±4.6); *Me* = 22	23.2 (±4.0); *Me* = 23	*Z* = −0.64, *p* = 0.523
TMT A time (baseline)	30.8 (±11.4); *Me* = 28	33.7 (±14.2); *Me* = 26	*Z* = −0.84, *p* = 0.403
TMT B time (baseline)	60.2 (±22.7); *Me* = 56	62.4 (±27.3); *Me* = 57	*Z* = −1.58, *p* = 0.115
DACOBS total (baseline)	161.8 (±26.7); *Me* = 164	161.6 (±30.1); *Me* = 163	*Z* = −0.12, *p* = 0.903
SOFAS I (baseline)	80.7 (±11.7); *Me* = 81	77.7 (±14.5); *Me* = 80	*Z* = −1.23, *p* = 0.219
SOFAS II (follow-up)	80.1 (±12.8); *Me* = 81	---	---
CAARMS total (baseline)	59.1 (±34.7); *Me* = 56.5	66.9 (±37.5); *Me* = 65	*Z* = −1.20, *p* = 0.231
CAARMS total (follow-up)	46.4 (±36.5); *Me* = 43	---	---

Note: *Me*—median, *Z*—Mann–Whitney’s U-test, *p*—*p*-value.

**Table 4:** The matrix of correlation for all quantitative variables (*n* = 134).

	TMT A	TMT B	DACOBS	SOFAS I	SOFAS II	CAARMS I	CAARMS II
TMT A (time)	---						
TMT B (time)	0.63 ***	---					
DACOBS (total score)	0.01	0.19 *	----				
SOFAS I (baseline)	−0.07	−0.18 *^a^	−0.39 ***	---			
SOFAS II (follow-up)	−0.21 *	−0.29 ***	−0.25 **	0.51 ***	---		
CAARMS I (baseline)	0.14	0.23 **	0.34 ***	−0.56 ***	−0.36 ***	---	
CAARMS II (follow-up)	0.12	0.21 *	0.14	−0.42 ***	−0.62 ***	0.49 ***	---

A probability note for *p*-values: * *p* ≤ 0.05, ** *p* ≤ 0.01, *** *p* ≤ 0.001. ^a^ insignificant after the controlling for multiple comparisons.

## Data Availability

The data presented in this study are available on request from the corresponding author. The data are not publicly available due to sensitive date it contain.

## References

[1] Kraepelin E. (1921). Manic depressive insanity and paranoia. J. Nerv. Ment. Disord..

[2] Van Os J., Hanssen M., Bijl R.V., Ravelli A. (2000). Strauss (1969) revisited: A psychosis continuum in the general population?. Schizophr. Res..

[3] Verdoux H., van Os J. (2002). Psychotic symptoms in non-clinical populations and the continuum of psychosis. Schizophr. Res..

[4] Giocondo J.G., Salum G.A., Gadelha A., Argolo F.C., Simioni A.R., Mari J.D., Miguel E.C., Bressan R.A., Pan P.M. (2021). Psychotic-Like Experiences and Common Mental Disorders in childhood and adolescence: Bidirectional and transdiagnostic associations in a longitudinal community-based study. Schizophr. Bull. Open.

[5] Kaymaz N., Drukker M., Lieb R., Wittchen H.U., Werbeloff N., Weiser M., Lataster T., van Os J. (2012). Do sub-threshold psychotic experiences predict clinical outcomes in unselected non-help-seeking population-based samples? A sys-tematic review and meta-analysis, enriched with new results. Psychol. Med..

[6] Healy C., Brannigan R., Dooley N., Coughlan H., Clarke M., Kelleher I., Cannon M. (2019). Childhood and adolescent psychotic experiences and risk of mental disorder: A systematic review and meta-analysis. Psychol. Med..

[7] Bhavsar V., Dorrington S., Morgan C., Hatch S.L., McGuire P., Fusar-Poli P., Mills J., MacCabe J.H., Hotopf M. (2021). Psychotic experiences, psychiatric comorbidity and mental health need in the general population: A cross-sectional and cohort study in Southeast London. Psychol. Med..

[8] Yates K., Lång U., Cederlöf M., Boland F., Taylor P., Cannon M., McNicholas F., DeVylder J.E., Kelleher I. (2019). Association of Psychotic Experiences With Subsequent Risk of Suicidal Ideation, Suicide Attempts, and Suicide Deaths: A Systematic Review and Meta-analysis of Longitudinal Population Studies. JAMA Psychiatry.

[9] Staines L., Healy C., Murphy F., Byrne J., Murphy J., Kelleher I., Cotter D., Cannon M. (2023). Incidence and Persistence of Psychotic Experiences in the General Population: Systematic Review and Meta- Analysis. Schizophr. Bull..

[10] Miller T.J., McGlashan T.H., Rosen J.L., Cadenhead K., Cannon T., Ventura J., McFarlane W., Perkins D.O., Pearlson G.D., Woods S.W. (2003). Prodromal assessment with the structured interview for prodromal syndromes and the scale of prodromal symptoms: Predictive validity, interrater reliability, and training to reliability. Schizophr. Bull..

[11] Yung A.R., McGorry P.D. (1996). The prodromal phase of first-episode psychosis: Past and current conceptualizations. Schizophr. Bull..

[12] Yung A.R., McGorry P.D. (1996). The initial prodrome in psychosis: Descriptive and qualitative aspects. Aust. N. Z. J. Psychiatry.

[13] Fusar-Poli P., Salazar de Pablo G., Correll C.U., Meyer-Lindenberg A., Millan M.J., Borgwardt S., Galderisi S., Bechdolf A., Pfennig A., Kessing L.V. (2020). Prevention of Psychosis: Advances in Detection, Prognosis, and Intervention. JAMA Psychiatry.

[14] Tandon N., Montrose D., Shah J., Rajarethinam R.P., Diwadkar V.A., Keshavan M.S. (2012). Early prodromal symptoms can predict future psychosis in familial high-risk youth. J. Psychiatr. Res..

[15] Van Os J., Linscott R.J., Myin-Germeys I., Delespaul P., Krabbendam L. (2009). A systematic review and meta-analysis of the psychosis continuum: Evidence for a psychosis proneness-persistence-impairment model of psychotic disorder. Psychol. Med..

[16] Nelson B., Fusar-Poli P., Yung A.R. (2012). Can we detect psychotic-like experiences in the general population?. Curr. Pharm. Des..

[17] Simon A.E., Grädel M., Cattapan-Ludewig K., Gruber K., Ballinari P., Roth B., Umbricht D. (2012). Cognitive functioning in at-risk mental states for psychosis and 2-year clinical outcome. Schizophr. Res..

[18] Fusar-Poli P., Deste G., Smieskova R., Barlati S., Yung A.R., Howes O., Stieglitz R.D., Vita A., McGuire P., Borgwardt S. (2012). Cognitive functioning in prodromal psychosis: A meta-analysis. Arch. Gen. Psychiatry.

[19] Giuliano A.J., Li H., Mesholam-Gately R.I., Sorenson S.M., Woodberry K.A., Seidman L.J. (2012). Neurocognition in the psychosis risk syndrome: A quantitative and qualitative review. Curr. Pharm. Des..

[20] Erlenmeyer-Kimling L., Rock D., Roberts S.A., Janal M., Kestenbaum C., Cornblatt B., Adamo U.H., Gottesman I.I. (2000). Attention, memory, and motor skills as childhood predictors of schizophrenia-related psychoses: The New York High-Risk Project. Am. J. Psychiatry.

[21] Woodberry K.A., Giuliano A.J., Seidman L.J. (2008). Premorbid IQ in schizophrenia: A meta-analytic review. Am. J. Psychiatry.

[22] Orellana G., Slachevsky A. (2013). Executive functioning in schizophrenia. Front. Psychiatry.

[23] Fatouros-Bergman H., Cervenka S., Flyckt L., Edman G., Farde L. (2014). Meta-analysis of cognitive performance in drug-naïve patients with schizophrenia. Schizophr. Res..

[24] Raffard S., Bayard S. (2012). Understanding the executive functioning heterogeneity in schizophrenia. Brain Cogn..

[25] Kuperberg G., Heckers S. (2000). Schizophrenia and cognitive function. Curr. Opin. Neurobiol..

[26] Breton F., Planté A., Legauffre C., Morel N., Adès J., Gorwood P., Ramoz N., Dubertret C. (2011). The executive control of attention differentiates patients with schizophrenia, their first-degree relatives and healthy controls. Neuropsychologia.

[27] Freedman D., Brown A.S. (2011). The developmental course of executive functioning in schizophrenia. Int. J. Dev. Neurosci. Off. J. Int. Soc. Dev. Neurosci..

[28] Bolt L.K., Amminger G.P., Farhall J., McGorry P.D., Nelson B., Markulev C., Yuen H.P., Schäfer M.R., Mossaheb N., Schlögelhofer M. (2019). Neurocognition as a predictor of transition to psychotic disorder and functional outcomes in ultra-high risk participants: Findings from the NEURAPRO randomized clinical trial. Schizophr. Res..

[29] Albert A.B., Abu-Ramadan T., Kates W.R., Fremont W., Antshel K.M. (2018). Childhood Executive Functioning Predicts Young Adult Outcomes in 22q11.2 Deletion Syndrome. J. Int. Neuropsychol. Soc. JINS.

[30] Remberk B., Bażyńska A.K., Krempa-Kowalewska A., Rybakowski F. (2014). Executive impairment predicts schizophrenia diagnosis and treatment status in mid-term follow-up of early-onset psychosis. Neuropsychobiology.

[31] Eslami A., Jahshan C., Cadenhead K.S. (2011). Disorganized symptoms and executive functioning predict impaired social functioning in subjects at risk for psychosis. J. Neuropsychiatry Clin. Neurosci..

[32] Snitz B.E., Macdonald A.W., Carter C.S. (2006). Cognitive deficits in unaffected first-degree relatives of schizophrenia patients: A meta-analytic review of putative endophenotypes. Schizophr. Bull..

[33] Ceaser A.E., Goldberg T.E., Egan M.F., McMahon R.P., Weinberger D.R., Gold J.M. (2008). Set-shifting ability and schizophrenia: A marker of clinical illness or an intermediate phenotype?. Biol. Psychiatry.

[34] Kumbhani S.R., Roth R.M., Kruck C.L., Flashman L.A., McAllister T.W. (2010). Nonclinical obsessive-compulsive symptoms and executive functions in schizophrenia. J. Neuropsychiatry Clin. Neurosci..

[35] Niendam T.A., Horwitz J., Bearden C.E., Cannon T.D. (2007). Ecological assessment of executive dysfunction in the psychosis prodrome: A pilot study. Schizophr. Res..

[36] Clark L.K., Warman D., Lysaker P.H. (2010). The relationships between schizophrenia symptom dimensions and executive functioning components. Schizophr. Res..

[37] Louise S., Gurvich C., Neill E., Tan E.J., Van Rheenen T.E., Rossell S. (2015). Schizotypal Traits are Associated with Poorer Executive Functioning in Healthy Adults. Front. Psychiatry.

[38] Kelleher I., Clarke M.C., Rawdon C., Murphy J., Cannon M. (2013). Neurocognition in the extended psychosis phenotype: Performance of a community sample of adolescents with psychotic symptoms on the MATRICS neurocognitive battery. Schizophr. Bull..

[39] Sheffield J.M., Karcher N.R., Barch D.M. (2018). Cognitive Deficits in Psychotic Disorders: A Lifespan Perspective. Neuropsychol. Rev..

[40] Kortte K.B., Horner M.D., Windham W.K. (2002). The Trail Making Test, part B: Cognitive flexibility or ability to maintain set?. Appl. Neuropsychol..

[41] Laere E., Tee S.F., Tang P.Y. (2018). Assessment of Cognition in Schizophrenia Using Trail Making Test: A Meta-Analysis. Psychiatry Investig..

[42] Hakamata Y., Matsui M., Tagaya H. (2014). Does neurocognitive function affect cognitive bias toward an emotional stimulus? Association between general attentional ability and attentional bias toward threat. Front. Psychol..

[43] Garety P.A., Bebbington P., Fowler D., Freeman D., Kuipers E. (2007). Implications for neurobiological research of cognitive models of psychosis: A theoretical paper. Psychol. Med..

[44] Rouy M., Saliou P., Nalborczyk L., Pereira M., Roux P., Faivre N. (2021). Systematic review and meta-analysis of metacognitive abilities in individuals with schizophrenia spectrum disorders. Neurosci. Biobehav. Rev..

[45] Roebers C.M. (2017). Executive function and metacognition: Towards a unifying framework of cognitive self-regulation. Dev. Rev..

[46] Flavell J. (1979). Metacognition and cognitive monitoring: A new area of cognitive developmental inquiry. Am. Psychol..

[47] Kleka P., Brycz H., Fanslau A., Pilarska A. (2019). Becoming aware of one’s own biases in emerging adulthood—A longitudinal study. Metacognitive approach. Adv. Cogn. Psychol..

[48] Ramos V.J., Torres M.L.M., Shen Y.-C. (2016). Cognitive Biases in Schizophrenia Spectrum Disorders. Schizophrenia Treatment—The New Facets.

[49] Dudley R., Taylor P., Wickham S., Hutton P. (2016). Psychosis, Delusions and the “Jumping to Conclusions” Reasoning Bias: A Systematic Review and Meta-analysis. Schizophr. Bull..

[50] So S.H., Siu N.Y., Wong H.L., Chan W., Garety P.A. (2016). ‘Jumping to conclusions’ data-gathering bias in psychosis and other psychiatric disorders—Two meta-analyses of comparisons between patients and healthy individuals. Clin. Psychol. Rev..

[51] Gawęda Ł., Moritz S., Ochoa S., So S.H. (2021). Editorial: The Relationship Between Cognitive Biases and Psychosis: Searching for Mechanisms. Front. Psychiatry.

[52] Díaz-Cutraro L., López-Carrilero R., García-Mieres H., Ferrer-Quintero M., Verdaguer-Rodriguez M., Barajas A., Grasa E., Pousa E., Lorente E., Barrigón M.L. (2022). The relationship between jumping to conclusions and social cognition in first-episode psychosis. Schizophrenia.

[53] Daalman K., Sommer I.E., Derks E.M., Peters E.R. (2013). Cognitive biases and auditory verbal hallucinations in healthy and clinical individuals. Psychol. Med..

[54] Gawęda Ł., Prochwicz K. (2015). A comparison of cognitive biases between schizophrenia patients with delusions and healthy individuals with delusion-like experiences. Eur. Psychiatry.

[55] Davies G., Fowler D., Greenwood K. (2017). Metacognition as a Mediating Variable Between Neurocognition and Functional Outcome in First Episode Psychosis. Schizophr. Bull..

[56] Moritz S., Krausz M., Gottwalz E., Lambert M., Perro C., Ganzer S., Naber D. (2000). Cognitive dysfunction at baseline predicts symptomatic 1-year outcome in first-episode schizophrenics. Psychopathology.

[57] Rajji T.K., Miranda D., Mulsant B.H. (2014). Cognition, function, and disability in patients with schizophrenia: A review of longitudinal studies. Can. J. Psychiatry Rev. Can. Psychiatr..

[58] Schubert K.O., Clark S.R., Baune B.T. (2015). The use of clinical and biological characteristics to predict outcome following First Episode Psychosis. Aust. N. Z. J. Psychiatry.

[59] Madjar N., Chubarov E., Zalsman G., Weiser M., Shoval G. (2019). Social skills, executive functioning and social engagement. Schizophr. Research. Cogn..

[60] Shim G., Kang D.H., Chung Y.S., Yoo S.Y., Shin N.Y., Kwon J.S. (2008). Social functioning deficits in young people at risk for schizophrenia. Aust. N. Z. J. Psychiatry.

[61] Meyer E.C., Carrión R.E., Cornblatt B.A., Addington J., Cadenhead K.S., Cannon T.D., McGlashan T.H., Perkins D.O., Tsuang M.T., Walker E.F. (2014). NAPLS group The relationship of neurocognition and negative symptoms to social and role functioning over time in individuals at clinical high risk in the first phase of the North American Prodrome Longitudinal Study. Schizophr. Bull..

[62] Tan B.L. (2009). Profile of cognitive problems in schizophrenia and implications for vocational functioning. Aust. Occup. Ther. J..

[63] Barkley R.A., Murphy K.R. (2010). Impairment in occupational functioning and adult ADHD: The predictive utility of executive function (EF) ratings versus EF tests. Arch. Clin. Neuropsychol. Off. J. Natl. Acad. Neuropsychol..

[64] Gilbert E., Marwaha S. (2013). Predictors of employment in bipolar disorder: A systematic review. J. Affect. Disord..

[65] Bryson G., Bell M.D. (2003). Initial and final work performance in schizophrenia: Cognitive and symptom predictors. J. Nerv. Ment. Dis..

[66] McGurk S.R., Mueser K.T., Harvey P.D., LaPuglia R., Marder J. (2003). Cognitive and symptom predictors of work outcomes for clients with schizophrenia in supported employment. Psychiatr. Serv..

[67] Lam M., Lee J., Rapisarda A., See Y.M., Yang Z., Lee S.A., Abdul-Rashid N.A., Kraus M., Subramaniam M., Chong S.A. (2018). Longitudinal Cognitive Changes in Young Individuals at Ultrahigh Risk for Psychosis. JAMA Psychiatry.

[68] Blanchard M.M., Jacobson S., Clarke M.C., Connor D., Kelleher I., Garavan H., Harley M., Cannon M. (2010). Language, motor and speed of processing deficits in adolescents with subclinical psychotic symptoms. Schizophr. Res..

[69] Bentall R., Fernyhough C., Morrison A., Lewis S., Corcoran R. (2007). Prospects for a cognitive-developmental account of psychotic experiences. Br. J. Clin. Psychol..

[70] Karcher N.R., Loewy R.L., Savill M., Avenevoli S., Huber R.S., Makowski C., Sher K.J., Barch D.M. (2022). Persistent and distressing psychotic-like experiences using adolescent brain cognitive development^SM^ study data. Mol. Psychiatry.

[71] Steenkamp L.R., Bolhuis K., Blanken L.M.E., Luijk M.P.C.M., Hillegers M.H.J., Kushner S.A., Tiemeier H. (2021). Psychotic experiences and future school performance in childhood: A population-based cohort study. J. Child Psychol. Psychiatry Allied Discip..

[72] Faragher E.B., Cass M., Cooper C.L. (2005). The relationship between job satisfaction and health: A meta-analysis. Occup. Environ. Med..

[73] Segrin C. (2019). Indirect Effects of Social Skills on Health Through Stress and Loneliness. Health Commun..

[74] Zubin J., Spring B. (1977). Vulnerability—A new view of schizophrenia. J. Abnorm. Psychol..

[75] Nuechterlein K.H., Dawson M.E. (1984). A heuristic vulnerability/stress model of schizophrenic episodes. Schizophr. Bull..

[76] Rudnick A., Lundberg E. (2012). The stress-vulnerability model of schizophrenia: A conceptual analysis and selective review. Curr. Psychiatry Rev..

[77] Leifker F.R., Bowie C.R., Harvey P.D. (2009). Determinants of everyday outcomes in schizophrenia: The influences of cognitive impairment, functional capacity, and symptoms. Schizophr. Res..

[78] Harvey P.D., Strassnig M.T., Silberstein J. (2019). Prediction of disability in schizophrenia: Symptoms, cognition, and self-assessment. J. Exp. Psychopathol..

[79] Wu C., Ye J., Li S., Wu J., Wang C., Yuan L., Wang H., Pan Y., Huang X., Zhong X. (2023). Predictors of everyday functional impairment in older patients with schizophrenia: A cross-sectional study. Front. Psychiatry.

[80] Hasmi L., Pries L.K., Ten Have M., de Graaf R., van Dorsselaer S., Bak M., Kenis G., Richards A., Lin B.D., O’Donovan M.C. (2021). What makes the psychosis ‘clinical high risk’ state risky: Psychosis itself or the co-presence of a non-psychotic disorder?. Epidemiol. Psychiatr. Sci..

[81] Rietdijk J., Hogerzeil S.J., van Hemert A.M., Cuijpers P., Linszen D.H., van der Gaag M. (2011). Pathways to psychosis: Help-seeking behavior in the prodromal phase. Schizophr. Res..

[82] Pionke-Ubych R., Frydecka D., Cechnicki A., Nelson B., Gawęda Ł. (2021). The Indirect Effect of Trauma via Cognitive Biases and Self-Disturbances on Psychotic-Like Experiences. Front. Psychiatry.

[83] Matheson S.L., Laurie M., Laurens K.R. (2023). Substance use and psychotic-like experiences in young people: A systematic review and meta-analysis. Psychol. Med..

[84] Halstead W.C. (1947). Brain and Intelligence.

[85] Reitan R.M. (1971). Trail Making Test results for normal and brain-damaged children. Percept. Und Mot. Ski..

[86] Lezak M.D. (1983). Neuropsychological Assessment.

[87] Arbuthnott K., Frank J. (2000). Trail making test, part B as a measure of executive control: Validation using a set-switching paradigm. J. Clin. Exp. Neuropsychol..

[88] Van der Gaag M., Schütz C., Ten Napel A., Landa Y., Delespaul P., Bak M., Tschacher W., de Hert M. (2013). Development of the Davos assessment of cognitive biases scale (DACOBS). Schizophr. Res..

[89] Gawęda Ł., Prochwicz K., Cella M. (2015). Cognitive biases mediate the relationship between temperament and character and psychotic-like experiences in healthy adults. Psychiatry Res..

[90] Goldman H.H., Skodol A.E., Lave T.R. (1992). Revising axis V for DSM-IV: A review of measures of social functioning. Am. J. Psychiatry.

[91] Samara M.T., Engel R.R., Millier A., Kandenwein J., Toumi M., Leucht S. (2014). Equipercentile linking of scales measuring functioning and symptoms: Examining the GAF, SOFAS, CGI-S, and PANSS. Eur. Neuropsychopharmacol. J. Eur. Coll. Neuropsychopharmacol..

[92] Magnani F., Amorosi S., Dell’Anna C., Lucarini V., Ballerini M., Marchesi C., Tonna M. (2023). The Inventory of Psychotic-Like Anomalous Self-Experiences (IPASE): An easy tool for investigating Self-Disorders, subjective experiences and global functioning. Eur. Psychiatry.

[93] Heinze K., Lin A., Nelson B., Reniers R.L.E.P., Upthegrove R., Clarke L., Roche A., Lowrie A., Wood S.J. (2018). The impact of psychotic experiences in the early stages of mental health problems in young people. BMC Psychiatry.

[94] Hilsenroth M.J., Ackerman S.J., Blagys M.D., Baumann B.D., Baity M.R., Smith S.R., Price J.L., Smith C.L., Heindselman T.L., Mount M.K. (2000). Reliability and validity of DSM-IV axis V. Am. J. Psychiatry.

[95] Rybarczyk B., Kreutzer J.S., DeLuca J., Caplan B. (2011). Social and Occupational Functioning Assessment Scale (SOFAS). Encyclopedia of Clinical Neuropsychology.

[96] Yung A.R., Pan Yuen H., Mcgorry P.D., Phillips L.J., Kelly D., Dell’olio M., Francey S.M., Cosgrave E.M., Killackey E., Stanford C. (2005). Mapping the Onset of Psychosis: The Comprehensive Assessment of At-Risk Mental States. Aust. N. Z. J. Psychiatry.

[97] Fusar-Poli P., Borgwardt S., Bechdolf A., Addington J., Riecher-Rössler A., Schultze-Lutter F., Keshavan M., Wood S., Ruhrmann S., Seidman L.J. (2013). The psychosis high-risk state: A comprehensive state-of-the-art review. JAMA Psychiatry.

[98] Yung A.R., Stanford C., Cosgrave E., Killackey E., Phillips L., Nelson B., McGorry P.D. (2006). Testing the Ultra High Risk (prodromal) criteria for the prediction of psychosis in a clinical sample of young people. Schizophr. Res..

[99] Jaracz J., Grzechowiak M., Raczkowiak L., Rataj K., Rybakowski J. (2012). Polish version of Comprehensive Assessment of At Risk Mental States (CAARMS)—The description of the method. Psychiatr. Pol..

[100] George D., Mallery P. (2016). IBM SPSS Statistics 23 Step by Step: A Simple Guide and Reference.

[101] Hayes A.F. (2018). Introduction to Mediation, Moderation, and Conditional Process Analysis: A Regression-Based Approach.

[102] Zhao X., Lynch J.G., Chen Q. (2010). Reconsidering Baron and Kenny: Myths and Truths about Mediation Analysis. J. Consum. Res..

[103] Frith C.D. (2014). The Cognitive Neuropsychology of Schizophrenia.

[104] Lysaker P.H., Leonhardt B.L., Pijnenborg M., van Donkersgoed R., de Jong S., Dimaggio G. (2014). Metacognition in schizophrenia spectrum disorders: Methods of assessment and associations with neurocognition, symptoms, cognitive style and function. Isr. J. Psychiatry Relat. Sci..

[105] Beck A.T. (1976). Cognitive Therapy and the Emotional Disorders.

[106] Beck A.T., Rector N.A., Stolar N., Grant P. (2009). Schizophrenia: Cognitive Theory, Research, and Therapy.

[107] Livet A., Navarri X., Potvin S., Conrod P. (2020). Cognitive biases in individuals with psychotic-like experiences: A systematic review and a meta-analysis. Schizophr. Res..

[108] Lee R.S., Hermens D.F., Redoblado-Hodge M.A., Naismith S.L., Porter M.A., Kaur M., White D., Scott E.M., Hickie I.B. (2013). Neuropsychological and socio-occupational functioning in young psychiatric outpatients: A longitudinal investigation. PLoS ONE.

[109] Oliver D., Reilly T.J., Baccaredda Boy O., Petros N., Davies C., Borgwardt S., McGuire P., Fusar-Poli P. (2020). What Causes the Onset of Psychosis in Individuals at Clinical High Risk? A Meta-analysis of Risk and Protective Factors. Schizophr. Bull..

[110] Martín-Santiago O., Suazo V., Rodríguez-Lorenzana A., Ruiz de Azúa S., Valcárcel C., Díez Á., Grau A., Domínguez C., Gallardo R., Molina V. (2016). Relaciones entre síntomas psicóticos subclínicos y rendimiento cognitivo en la población general [Relationship between subclinical psychotic symptoms and cognitive performance in the general population]. Rev. Psiquiatr. Salud Ment..

[111] Miley K., Hadidi N., Kaas M., Yu F. (2020). Cognitive Training and Remediation in First-Episode Psychosis: A Literature Review. J. Am. Psychiatr. Nurses Assoc..

[112] Rammos A., Sullivan S.A., Kounali D., Jones H.J., Hammerton G., Hines L.A., Zammit S. (2022). Precursors and correlates of transient and persistent longitudinal profiles of psychotic experiences from late childhood through early adulthood. Br. J. Psychiatry.

[113] Muddle S., Jones B., Taylor G., Jacobsen P. (2022). A systematic review and meta-analysis of the association between emotional stress reactivity and psychosis. Early Interv. Psychiatry.

[114] DeLuca J.S., Rakhshan Rouhakhtar P., Klaunig M.J., Akouri-Shan L., Jay S.Y., Todd T.L., Sarac C., Andorko N.D., Herrera S.N., Dobbs M.F. (2022). Psychosis-like experiences and resilience: A systematic and critical review of the literature. Psychol. Serv..

[115] Prochwicz K., Kłosowska J., Dembińska A. (2020). The Mediating Role of Stress in the Relationship between Attention to Threat Bias and Psychotic-Like Experiences Depends on Coping Strategies. Front. Psychiatry.

[116] Vines L., Bridgwater M., Bachman P., Hayes R., Catalano S., Jalbrzikowski M. (2022). Elevated emotion reactivity and emotion regulation in individuals at clinical high risk for developing psychosis and those diagnosed with a psychotic disorder. Early Interv. Psychiatry.

[117] Ader L., Schick A., Simons C., Delespaul P., Myin-Germeys I., Vaessen T., Reininghaus U. (2022). Positive Affective Recovery in Daily Life as a Momentary Mechanism across Subclinical and Clinical Stages of Mental Disorder: Experience Sampling Study. JMIR Ment. Health.

[118] Bae S.M., Lee S.H., Park Y.M., Hyun M.H., Yoon H. (2010). Predictive factors of social functioning in patients with schizophrenia: Exploration for the best combination of variables using data mining. Psychiatry Investig..

[119] De Winter L., Couwenbergh C., van Weeghel J., Hasson-Ohayon I., Vermeulen J.M., Mulder C.L., Boonstra N., Klaver K.M., Oud M., de Haan L. (2022). Changes in social functioning over the course of psychotic disorders-A meta-analysis. Schizophr. Res..

[120] García-Portilla M.P., García-Álvarez L., González-Blanco L., Dal Santo F., Bobes-Bascarán T., Martínez-Cao C., García-Fernández A., Sáiz P.A., Bobes J. (2021). Real-World Functioning in Patients with Schizophrenia: Beyond Negative and Cognitive Symptoms. Front. Psychiatry.

[121] Yang Z., Lee S.H., Abdul Rashid N.A., See Y.M., Dauwels J., Tan B.L., Lee J. (2021). Predicting Real-World Functioning in Schizophrenia: The Relative Contributions of Neurocognition, Functional Capacity, and Negative Symptoms. Front. Psychiatry.

[122] Romanowska S., Best M.W., Bowie C.R., Depp C.A., Patterson T.L., Penn D.L., Pinkham A.E., Harvey P.D. (2022). Examining the association of life course neurocognitive ability with real-world functioning in schizophrenia-spectrum disorders. Schizophr. Research. Cogn..

[123] Hajdúk M., Penn D.L., Harvey P.D., Pinkham A.E. (2021). Social cognition, neurocognition, symptomatology, functional competences and outcomes in people with schizophrenia—A network analysis perspective. J. Psychiatr. Res..

[124] Van der Steen Y., Myin-Germeys I., van Nierop M., Ten Have M., de Graaf R., van Dorsselaer S., van Os J., van Winkel R. (2019). ‘False-positive’ self-reported psychotic experiences in the general population: An investigation of outcome, predictive factors and clinical relevance. Epidemiol. Psychiatr. Sci..

[125] Remberk B. (2017). Clinical significance of psychotic-like experiences in children and adolescents. Psychiatr. Pol..

[126] Yung A.R., Buckby J.A., Cotton S.M., Cosgrave E.M., Killackey E.J., Stanford C., Godfrey K., McGorry P.D. (2006). Psychotic-like experiences in nonpsychotic help-seekers: Associations with distress, depression, and disability. Schizophr. Bull..

[127] Yung A.R., Nelson B., Baker K., Buckby J.A., Baksheev G., Cosgrave E.M. (2009). Psychotic-like experiences in a community sample of adolescents: Implications for the continuum model of psychosis and prediction of schizophrenia. Aust. N. Z. J. Psychiatry.

[128] Armando M., Nelson B., Yung A.R., Saba R., Monducci E., Dario C., Righetti V., Birchwood M., Fiori Nastro P., Girardi P. (2012). Psychotic experience subtypes, poor mental health status and help-seeking behaviour in a community sample of young adults. Early Interv. Psychiatry.

[129] Armando M., Nelson B., Yung A.R., Ross M., Birchwood M., Girardi P., Fiori Nastro P. (2010). Psychotic-like experiences and correlation with distress and depressive symptoms in a community sample of adolescents and young adults. Schizophr. Res..

[130] Barragan M., Laurens K.R., Navarro J.B., Obiols J.E. (2011). Psychotic-like experiences and depressive symptoms in a community sample of adolescents. Eur. Psychiatry J. Assoc. Eur. Psychiatr..

[131] Yung A.R., Buckby J.A., Cosgrave E.M., Killackey E.J., Baker K., Cotton S.M., McGorry P.D. (2007). Association between psychotic experiences and depression in a clinical sample over 6 months. Schizophr. Res..

[132] Sullivan S.A., Kounali D., Cannon M., David A.S., Fletcher P.C., Holmans P., Jones H., Jones P.B., Linden DE J., Lewis G. (2020). A Population-Based Cohort Study Examining the Incidence and Impact of Psychotic Experiences From Childhood to Adulthood, and Prediction of Psychotic Disorder. Am. J. Psychiatry.

[133] Karcher N.R. (2022). Psychotic-like experiences in childhood and early adolescence: Clarifying the construct and future directions. Schizophr. Res..

